# Acute kidney injury after COVID-19 vaccines: correspondence

**DOI:** 10.1080/0886022X.2022.2091457

**Published:** 2022-06-22

**Authors:** Rujittika Mungmunpuntipantip, Viroj Wiwanitkit

**Affiliations:** a Private Academic Consultant, Bangkok, Thailand; b Joseph Ayobabalola University, Ikeji-Arakeji, Nigeria

Dear Editor,

We would like to share ideas on “Acute kidney injury (AKI) after COVID-19 vaccines: a real-world study [1].” Luo et al. discovered that AKI could arise following the COVID-19 vaccinations, most notably in older people. However, the causality must be determined further. While the COVID-19 immunization is useful, we are all afraid that it may possibly be dangerous. In the current study, AKI, immunization participants may have side effects. However, no conclusions can be reached because there is no pre-vaccination information on the health and immunological status of vaccine recipients. A comorbidity in the patient could be the source of the problem [2]. Vaccination recipients may develop co-infections after immunization, which are misconstrued as a bad consequence. Dengue fever, for example, can occur concurrently [2] and cause AKI [3]. Furthermore, Luo et al. did not exclude patients with active COVID-19 infection or those who had recently been infected with COVID-19, and there is still the possibility of asymptomatic COVID-19, a common clinical problem [4], which could be another confounding factor in the observed post vaccination adverse event.
